# Complete mitochondrial genome of the mangrove killifish *Kryptolebias hermaphroditus* (Cyprinodontiformes, Rivulidae)

**DOI:** 10.1080/23802359.2016.1197075

**Published:** 2016-07-23

**Authors:** Hui-Su Kim, Dae-Sik Hwang, Atsushi Hagiwara, Yoshitaka Sakakura, Jae-Seong Lee

**Affiliations:** a Department of Biological Science, College of Science, Sungkyunkwan University, Suwon, South Korea;; b Graduate School of Fisheries and Environmental Sciences, Nagasaki University, Nagasaki, Japan

**Keywords:** Complete mitochondrial genome, fish, *Kryptolebias hermaphroditus*, mangrove killifish

## Abstract

The complete mitochondrial genome was sequenced from the mangrove killifish (*Kryptolebias hermaphroditus*). The genome sequence was 17,487 bp in size, and the gene order and contents were identical with those of the congeneric species (*K. marmoratus*) in the genus *Kryptolebias* with emphasis on the second control region (795 bp). Of 13 protein-coding genes (PGCs), 5 genes (*ND2*, *CO2*, *CO3*, *ND3*, and *Cytb*) had incomplete stop codons as shown in *K. marmoratus*. Furthermore, the stop codon of *ND6* gene was AGG, while the start codon of *CO1* gene was GTG. The base composition of *K. hermaphroditus* mitogenome showed an anti-G bias (13.45% and 8.19%) on the second and third position of the protein-coding genes (PCGs), respectively.

In the genus *Kryptolebias*, eight species are described (Rainer & Pauly 2012). As a valuable model for experimental research, the genetic compositions of laboratory stocks were analyzed in the self-fertilizing fish *K. marmoratus* (Tatarenkov et al. 2010). After these analyses, the PAN-RS strain of *K. marmoratus* was renamed as *K. hermaphroditus*, which is also self-fertilizing (Kanamori et al. 2016). Intraspecific nucleotide variation of the complete mitogenome was 0.23% between two strains of *K. marmoratus* (Tatarenkov et al. 2015). In this paper, we report the complete mitochondrial genome of the mangrove killifish (*Kryptolebias hermaphroditus*) to better understand the phylogenetic position within the genus *Kryptolebias*.

The specimen used for our laboratory stocks was collected near Bocas del Toro, Republic of Panama in 1994 (kindly provided by Dr. W.P. Davis of the U.S. Environmental Protection Agency, USA) and maintained at the Laboratory of Professor Yoshitaka Sakakura, Nagasaki University in Japan. The specimen was deposited in the ichthyological collection of the Faculty of Fisheries, Nagasaki University (FFNU) under the accession no. FFNU-P-2087. We sequenced the complete genome of the mangrove killifish *K. hermaphroditus* from liver genomic DNA of *K. hermaphroditus* with a 300 bp paired-end library using the Illumina HiSeq 2000 platform (GenomeAnalyzer, Illumina, San Diego, CA). *De novo* assembly was conducted using Ray assembler 2.3.2-devel (http://denovoassembler.sourceforge.net/). Of the assembled 64,930 *K. hermaphroditus* contigs, three contigs were mapped to the mitochondrial DNA of *Kryptolebias marmoratus* (GenBank No. AF283503). Reference-guided assembly with three mapped contigs was conducted using Geneious R9 (Biomatters, http://www.geneious.com/). As a result, a single mitochondrial genome was obtained with full length.

The complete mitochondrial genome of *K. hermaphroditus* was 17,487 bp (GenBank accession no. KX268503). The direction of genes was identical with those of a congeneric species (*K. marmoratus*) of the genus *Kryptolebias*, including the presence of the second control region that does not exist in other fishes (Lee et al. 2001; Tatarenkov et al. 2015). Between two sister species (*K. hermaphroditus*, *K. marmoratus*), the similarities of amino acids and nucleotides of 13 PCGs were 97.5% (94.54% for *ND2* and 99.81% for *CO1*) and 96.18% (93.92% for *ND4* and 97.68% for *CO2*), respectively. Of the 13 PCGs, 5 genes (*ND2*, *CO2*, *CO3*, *ND3*, and *Cytb*) had incomplete stop codons, as shown in *K. marmoratus*. Particularly, in *K. hermaphroditus*, there is an anti-G bias (13.45% and 8.19%) at the second and third position of the codons. *Kryptolebias hermaphroditus* used GTG as a start codon of the *CO1* gene, similar to other vertebrate mitochondrial genomes, and used AGG as a stop codon for *ND6* gene. The mitochondrial genome base composition of 13 PCGs was 24.65% for A, 31.71% for T, 15.61% for G and 28.03% for C. The A + T base composition (56.36%) was slightly higher than G + C (43.64%).

The placement of *K. hermaphroditus* among all cyprinodontiform fishes with known complete mitogenomes is shown in Figure 1. *Kryptolebias hermaphroditus* clustered closely to the congener with known mitogenome, *K. marmoratus*, and diverged more recently.

**Figure 1.**
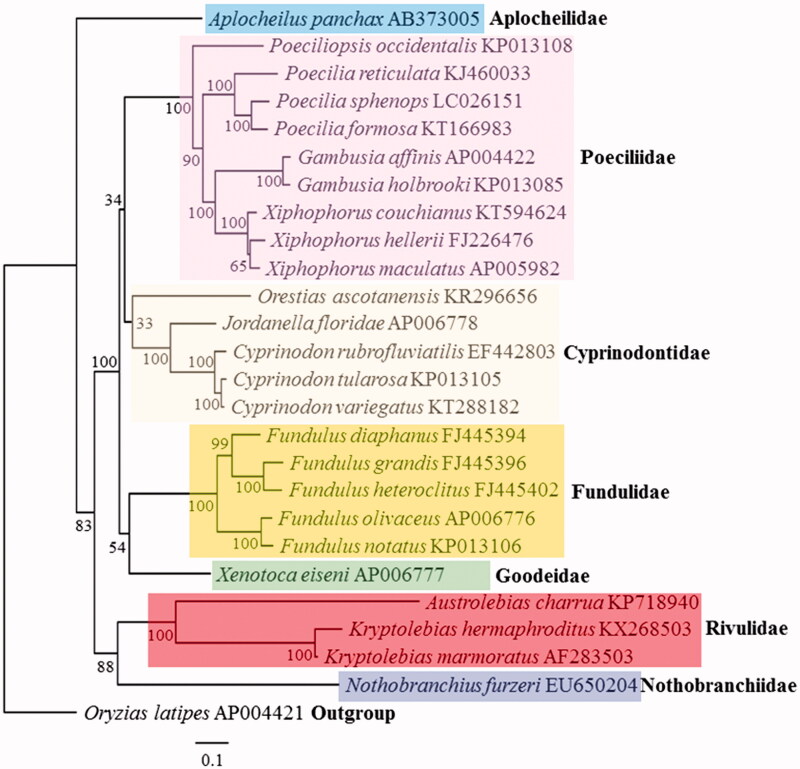
Phylogenetic analysis. We compared the mitochondrial genomes of 25 cyprinodontiform fishes. Thirteen protein-coding genes of each mt genomes were aligned by ClustalW and edited manually. Maximum likelihood (ML) analysis was performed by Raxml 8.2.8 (http://sco.h-its.org/exelixis/software.html) with GTR + Gamma + I nucleotide substitution model. The rapid bootstrap analysis was conducted with 10,000 replications with 48 threads running in parallel and the bootstrap value was on the clade. Outgroup was Japanese medaka (*Oryzias latipes*).
